# Immune cell infiltration characteristics and related core genes in lupus nephritis: results from bioinformatic analysis

**DOI:** 10.1186/s12865-019-0316-x

**Published:** 2019-10-21

**Authors:** Yiling Cao, Weihao Tang, Wanxin Tang

**Affiliations:** 1 Department of Nephrology, West China Hospital, Sichuan University, No.37, Guoxue alley, Chengdu, 610000 Sichuan China; 2 Chengdu Foreign Language School, Chengdu, Sichuan China

**Keywords:** Systemic lupus erythematosus, Lupus nephritis, CIBERSORT, GSEA, Immune infiltration

## Abstract

**Background:**

Lupus nephritis (LN) is a common complication of systemic lupus erythematosus that presents a high risk of end-stage renal disease. In the present study, we used CIBERSORT and gene set enrichment analysis (GSEA) of gene expression profiles to identify immune cell infiltration characteristics and related core genes in LN.

**Results:**

Datasets from the Gene Expression Omnibus, GSE32591 and GSE113342, were downloaded for further analysis. The GSE32591 dataset, which included 32 LN glomerular biopsy tissues and 14 glomerular tissues from living donors, was analyzed by CIBERSORT. Different immune cell types in LN were analyzed by the Limma software. Gene Ontology (GO) and Kyoto Encyclopedia of Genes and Genomes (KEGG) pathway analysis based on GSEA were performed by clusterProfiler software. Lists of core genes were derived from Spearman correlation between the most significant GO term and differentially expressed immune cell gene from CIBERSORT. GSE113342 was employed to validate the association between selected core genes and clinical manifestation. Five types of immune cells revealed important associations with LN, and monocytes emerged as having the most prominent differences. GO and KEGG analyses indicated that immune response pathways are significantly enriched in LN. The Spearman correlation indicated that 15 genes, including FCER1G, CLEC7A, MARCO, CLEC7A, PSMB9, and PSMB8, were closely related to clinical features.

**Conclusions:**

This study is the first to identify immune cell infiltration with microarray data of glomeruli in LN by using CIBERSORT analysis and provides novel evidence and clues for further research of the molecular mechanisms of LN.

## Background

Systemic lupus erythematosus (SLE), one of the most complicated autoimmune diseases in the world, is caused by various endogenous antigens [1]. Lupus nephritis (LN), a common and serious complication of SLE, is characterized by hematuria, proteinuria, and impaired glomerular filtration rate [2]. The lack of understanding regarding the molecular mechanisms of LN hinders the development of specific targeted therapy for this progressive disease [3]. Tracking the biological changes in LN at the genomic level is a worthwhile strategy [4]. In recent years, gene sequencing technology combined with bioinformatic analysis has been conducted to identify genes relevant to diseases that might serve as prognostic biomarkers and be developed as therapeutic targets in the future [5]. Bioinformatic analysis can process large amounts of samples within an extremely short time and provide valuable information about diseases, and several genes closely associated with SLE have been identified and driven research innovations in recent years [6–8]. However, few studies utilized bioinformatic analysis to characterize kidney tissue in the context of LN.

Many previous works found that immune cell infiltration is associated with treatment and clinical outcome in different types of cancer [9, 10]. Immune cells consisting of innate and adaptive immune populations, including dendritic cells, macrophages, neutrophils, T cells, and B cells, are associated with active and suppressive immune functions [11]. However, given the functionally distinct cell types that comprise the immune response, assessing immune infiltration and determining whether differences in the composition of the immune infiltration can improve the development of novel immunotherapeutic drugs to target these cells is important. The CIBERSORT algorithm is an analytical tool whereby RNA-seq data can be used to assess the expression changes of immune cells and obtain the proportion of various types of immune cells from the samples. CIBERSORT offers 22 cell types encompassing monocytes, natural killer cells, B cells, T cells, eosinophils, macrophages, neutrophils, plasma cells, dendritic cells, and mast cells [12]. It has been prevalently used to determine the immune cell landscapes in many malignant tumors such as breast cancer, hepatocellular carcinoma, and colorectal cancer [13–15]. In SLE pathogenesis, various immune cells have been widely evaluated and demonstrated to be harmful [16]. Immune cell infiltration is also a hallmark of LN. Immune cells, such as monocytes, B cells, and T cells, are recruited to kidney tissue and produce cytokines and chemokines to cause tissue damage [17]. However, the landscape of immune infiltration in LN has not been entirely revealed.

Although LN can affect all components of the kidney, the glomerulus is the most suitable tissue and is closely related to the pathogenesis and treatment of the disease [18]. In our present study, the microarray data were downloaded from the Gene Expression Omnibus (GEO) database. By using CIBERSORT, we first investigated the difference in immune infiltration between LN kidney tissue and normal tissue in 22 subpopulations of immune cells. Gene set enrichment analysis (GSEA) was employed for functional enrichment analyses and to determine the most significant functional terms. A list of genes closely related to immune infiltration was screened out and validated against another dataset with clinical information from the GEO database. This study aimed to describe the characteristics of LN glomerular immune infiltration for the first time and to identify some key genes related to immune infiltration that affect clinical manifestation, so as to provide data resources for future research.

## Results

### Bioinformatic analysis workflows and data description

**Fig. 1:**
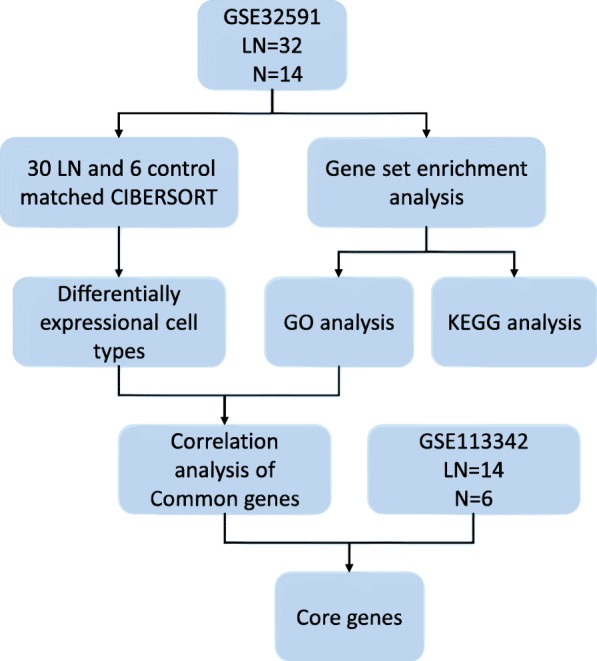
Workflows of our bioinformatic analysis

Our workflows are shown in Fig. 1. We first investigated the difference of immune cell infiltration between normal glomerular tissues and LN glomerular tissues. Next, we discovered the most significant GO and KEGG functional term by GSEA. We screened out a list of genes closely related to immune infiltration and validated these genes against the clinical data. A total of 46 samples from GSE32591 were used in this study, including 32 LN glomerular biopsy tissues and 14 glomerular tissues from living donors. After data processing, the expression matrix of 30 LN glomerular samples and 6 normal control glomerular samples was obtained by screening the immune cell infiltration. GSE113342 contained 14 biopsy kidney tissues and 6 normal tissues.

### Performance of CIBERSORT

**Fig. 2:**
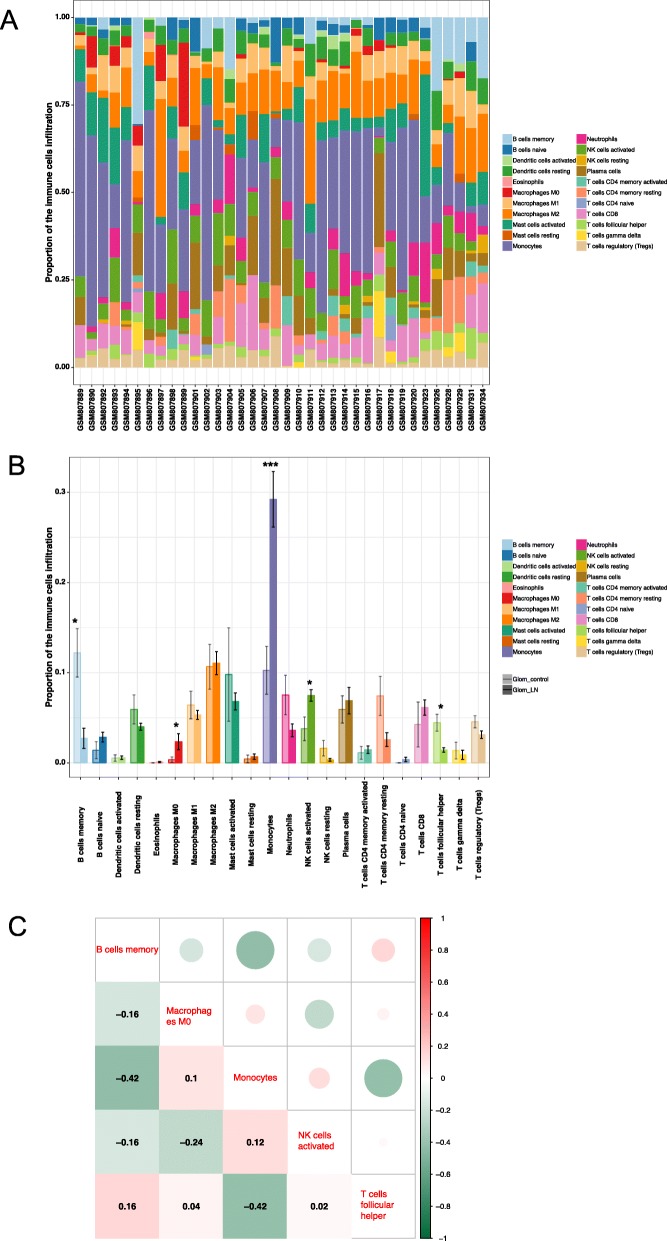
Landscape of immune infiltration in LN. **a**. Bar charts of 22 immune cell proportions in LN and normal tissues. **b**. Differential expression of different types of immune cells between LN and normal tissues. **c**. Correlation matrix of five types of immune cell proportions. Variables are ordered by matrix heat map. Data was collated by using R package tidyverse (version 1.2.1). R package ggpubr (version 0.1.8) was used for T test. Results visualization was performed by using R package ggplot2 (version 3.1.0). Correlation analysis and visualization were performed by using R package corrplot (version 0.84)

Figure 2a shows the proportions of immune cells in 36 kidney tissues. Obviously, monocytes accounted for the majority of all infiltrating cells, especially in LN tissue. The differential expressional proportion of immune infiltration cells in the LN and control groups is shown in Fig. 2b. Five types of immune cells, namely, memory B cells, M0 macrophages, monocytes, activated NK cells, and follicular helper T (Tfh) cells, were differentially expressed. Monocytes, M0 macrophages, and activated NK cells were upregulated in LN tissue. The *P*-values of the five types of immune cells were 0.014, 0.04, 0.0001, 0.037, and 0.022, respectively. Among them, the increase in monocytes was the most significant. Memory B cells and Tfh cells were downregulated. Figure 2c indicates the correlation between these differentially expressed types of immune cells. The five types of immune cells were weakly to moderately correlated. Monocytes were negatively correlated with memory B cells and Tfh cells (r = − 0.42 and r = − 0.42, respectively), which indicated that the function of monocytes, Tfh cells, and memory B cells in LN may be antagonistic. However, the relationship between memory B cells and Tfh cells was synergistic.

### GSEA-based GO analysis

**Fig. 3:**
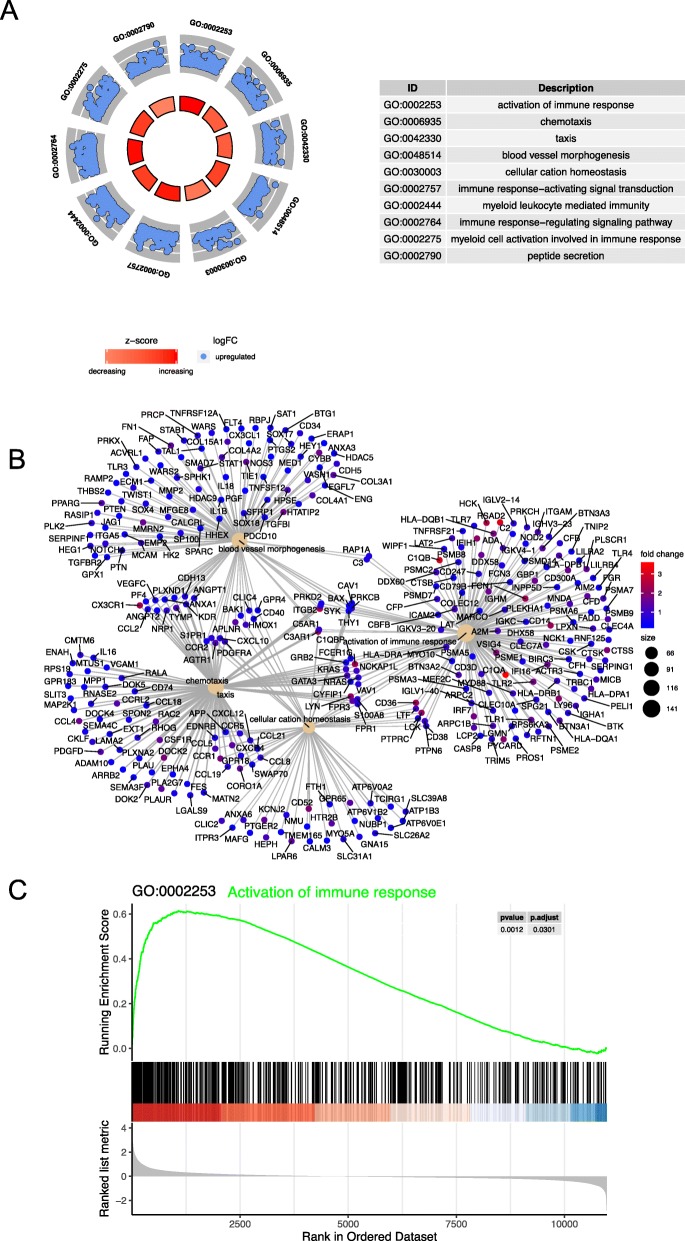
GO analysis and GSEA. **a**. Significantly enriched GO biological processes of genes. The blue dots in the graph mean upregulated gene. The depth of the inner arc area shows decrease or increase of the biological process. **b**. Gene correlation between most prominent GO terms. The depth of the color represents the fold change of gene. The area of circle means gene counts. **c**. GSEA-based GO analysis-enrichment plots of representative gene sets: activation of immune response. The green line means enrichment profile. GO and GSEA analysis was performed by using R package clusterProfiler (version 3.8.1); R package DOSE (version 3.6.1); and R package org. Hs.eg.db (version 3.6.0). The analysis results were visualized by using R package Enrichplot (version 1.2.0) and R package GOplot (version 1.0.2)

On the basis of the GO biological process, the top 10 most significantly enriched GO terms are presented in Fig. 3a. Genes in GO terms were primarily associated with “activation of immune response (GO:0002253),” “chemotaxis (GO:0006935),” and “taxis (GO:0042330).” A total of 478 genes were involved in “activation of immune response.” These results confirmed that immune response is very important in LN. Our GO analysis presented numerous important genes associated with this function. The details of GO analysis are shown in Additional file 1: Table S1.

The connection between the most prominent GO terms is shown in Fig. 3b. The network-presented numerous genes, such as RSAD2, C1QA, C1QB, CX3CR1, ITGB2, FCER1G, and CCR1, that were significantly differentially expressed in LN. Moreover, ITGB2, FCER1G, C5AR1, LYN, CD36, and PTPRC were important bridge genes between different biological processes. We used all of the “activation of immune response” gene sets for GSEA, and the gene set enrichment result is presented in Fig. 3c. The enrichment showed that the gene set was enriched at the front of the sequence (ES = 0.61). Over 100 genes were core genes that increased during this process. We obtained the list of all core genes, such as C1QA, RSAD2, C1QB, ITGB2, HCK, C3AR1, FCN1 and FCER1G, for subsequent analysis.

### GSEA-based KEGG analysis

**Fig. 4:**
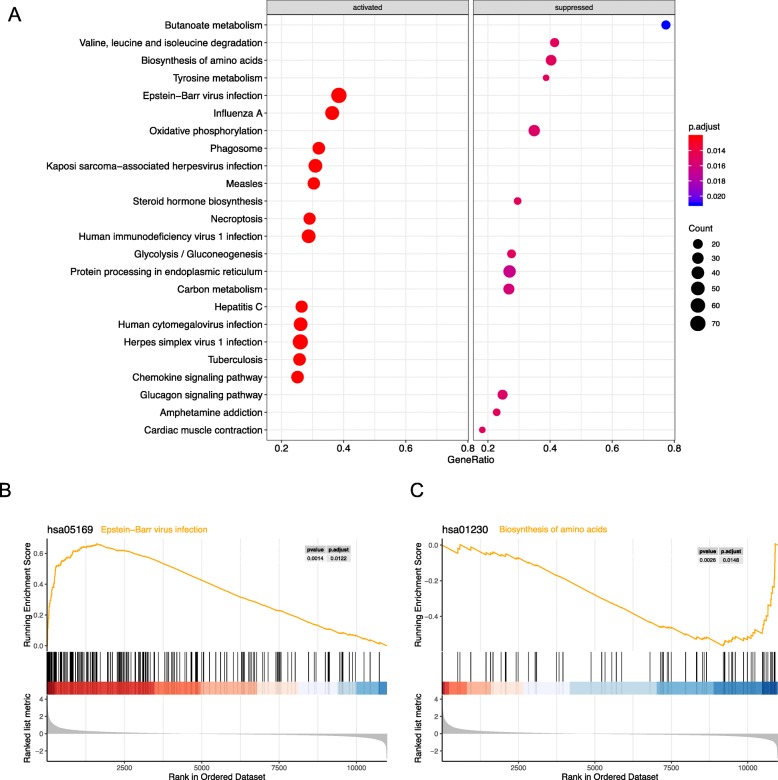
KEGG and GSEA. **a**. Significantly enriched activated and suppressed KEGG pathways. The vertical items are the names of KEGG terms, and the length of horizontal graph represents the gene ratio. The depth of the color represents the adjusted *p*-value. The area of circle in the graph means gene counts. **b**. GSEA-based KEGG-enrichment plots of representative gene sets from activated pathway: Epstein–Barr virus infection. **c**. GSEA-based KEGG-enrichment plots of representative gene sets from suppressed pathway: Biosynthesis of amino acids. KEGG and GSEA analysis was performed by using R package clusterProfiler (version 3.8.1); R package DOSE (version 3.6.1); and R package org. Hs.eg.db (version 3.6.0). The analysis results were visualized by using R package Enrichplot (version 1.2.0)

A total of 24 prominent KEGG pathways including activated and suppressed pathways were selected (Fig. 4a). Activated pathways, such as “Epstein–Barr virus infection,” “Herpes simplex virus 1 infection,” “Influenza A,” “Human cytomegalovirus infection,” and “Kaposi sarcoma-associated herpesvirus infection,” were related to cellular immunity against viral infection. The result indicated that the activation of signaling pathways in LN is similar to that of viral infection. However, suppressed pathways were mainly concentrated on metabolic process, such as “Biosynthesis of amino acids,” “Valine, leucine and isoleucine degradation,” “Steroid hormone biosynthesis,” and “Oxidative phosphorylation.”

GSEA enrichment plots of representative gene sets on “Epstein–Barr virus infection” and “Biosynthesis of amino acids” are shown in Fig. 4b and 4c, respectively. In the activated pathway, 182 genes participated in the EB virus infection pathway and were concentrated at the front of the sequence. The core genes such as ISG15, OAS1, OAS2, OAS3, LYN, HLA-DQB1, and TLR2 were upregulated. In the suppressed pathway, only 62 functional genes were involved and were enriched at the back of the sequence.

### Discovery of core genes

**Fig. 5:**
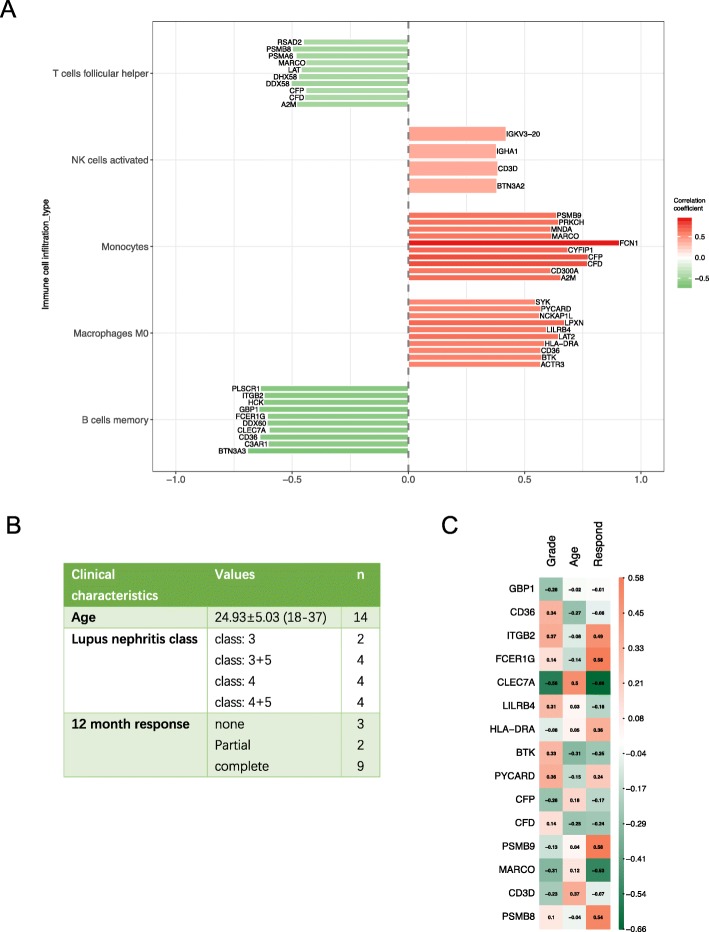
Common core genes and correlation with clinical characteristics. **a**. Correlation analysis between core genes in activation of immune response and five types of immune infiltrating cells. The vertical items are the names of immune cells. The horizontal items indicate the correlation coefficient. Red represents positive correlation, whereas green represents negative correlation. **b**. Summary of clinical information in LN group from GSE32591 dataset. Clinical characteristics are age, grade, and treatment response. **c**. Analysis of the correlation between intersecting genes from activation of immune response with immune infiltrating cells and three clinical characteristics. Correlation analysis was performed by using R package Hmisc (version 4.1.1). The results were visualized by using R package ggplot2 (version 3.1.0)

The correlation between core genes came from the GSEA GO term “activation of immune response” and five types of immune infiltrating cells are shown in Fig. 5a. A total of 44 genes showed close connection with immune infiltrating cells. Genes such as RSAD2, PSMB8, PSMA6, and MARCO were negatively correlated with Tfh cells. PLSCR1, ITGB2, HCK, and GBP1 were negatively related to memory B cells. FCN1, PSMB9, PRKCH, and A2M were positively correlated with monocytes. SYK, PYCARD, LPXN, and BTK were positively related to M0 macrophages. However, our analysis only found four genes correlated with activated NK cells.

### Validation of core genes

Figure 5b shows the clinical information of GSE113342. The LN grade was mainly concentrated on 3–5 classes. The core gene list was validated in the clinical dataset. Grade, age, and 12-month response were chosen as clinical indicators (Fig. 5c). Through Spearman correlation analysis between core gene list and clinical information, GBP1, CD36, ITGB2, FCER1G, CLEC7A, LILRB4, HLA-DRA, BTK, PYCARD, CFP, CFD, PSMB9, MARCO, CD3D, and PSMB8 were found to be active in both networks, which indicated that these core genes were associated with immune infiltration and affected clinical manifestation. Among them, CLEC7A was positively correlated with age (r = 0.5) but negatively correlated with grade and 12-month response (r = − 0.56 and r = − 0.66, respectively). MARCO was negatively correlated with treatment response (r = − 0.53). FCER1G, PSMB8, and PSMB9 were positively correlated with treatment response (r = 0.58, r =  0.58, and r =  0.54, respectively).

## Discussion

With the development of bioinformatics, increasing attention has been focused on finding hub genes in various diseases, and the collected information on these genes can provide new means for exploring diseases. Multiple susceptibility genes may determine disease occurrence.

In this study, we uncovered different expressional cell patterns of immune infiltration in LN and association with clinical features. Monocytes were the prominent differentially expressed cells. These are important components of the innate immune system; they have an antigen presentation capacity and produce several inflammatory cytokines in SLE [19]. Monocytes accounted for approximately 4% of blood leukocytes in healthy mice and over 50% in lupus-prone mice [20]. Our result also showed that monocytes constituted 30–50% of immune cells in human LN glomeruli. Activated NK cells were also increased in glomeruli. However, reports from other studies showed lower proportions of NK cells in SLE patient blood, especially in patients with LN [21, 22]. However, in rheumatoid arthritis tissue, NK cells were reported to contradict the function of circulating NK cells, which indicated that tissue NK cells may have different effects as compared with blood NK cells in autoimmune disease [23]. Clinical and experimental evidence indicated that aberrant memory B cells and Tfh cells played an important role in the pathogenesis of human SLE [24–26]. Resting M0 macrophages can polarize into M1 and M2 macrophages in the presence of the appropriate cytokines [27]. However, no research has explained the function of increased M0 macrophages in LN. The specific role of these immune cells in functional immune responses still remains to be elucidated.

“Activation of immune response” was the top associated pathway under GSEA-based GO analysis. The activation of innate and adaptive immune system triggering immune complex deposition, complement activation, and self-antigen production displayed a toxic effect on renal glomerular and tubular cells, thereby promoting the development of nephritis in patients with SLE [28, 29]. Through KEGG pathway analysis, several kinds of virus infection pathways were associated with LN. The immunoreaction of LN and response to virus may share several common features.

By combining CIBERSORT results and “activation of immune response” GO term, we found many novel commonly expressed genes, some of which were important in autoimmune diseases. For example, FCN1 was proven to be associated with monocytes in patients with microscopic polyangiitis [30]. Another study involving weighted correlation network analysis showed that RSAD2 related to CD4+ T cells may be the most highly ranked hub gene in SLE [7]. BTK mediates TLR signaling in macrophages and may be a promising treatment approach for LN [31–33]. These genes were observed to be highly or mildly associated with immune cells in kidney tissues.

Through a review of documents about lupus and related genes [34–48], 15 core genes related to clinical manifestation were found to be associated in autoimmune disease (Table 1). FCER1G, CLEC7A, MARCO, CLEC7A, PSMB9, and PSMB8 showed apparent correlation with clinical manifestation. FCER1G, which is associated with multiple leukocyte receptor complexes and mediates signal transduction, plays a negative regulatory role in the B cell responses [36]. CLEC7A, also known as dectin-1, is a type II membrane receptor expressed in the membrane of some leukocytes and likely contributes to the synthesis of pro-inflammatory cytokines in autoimmune conditions [37]. MARCO, a scavenger receptor family, plays important roles in the clearance of apoptotic cells. The presence of anti-MARCO antibodies in SLE patients might contribute to the breakdown of self-tolerance and the pathogenesis of SLE [46]. PSMB8 is involved in antigen processing and presentation in naïve CD4+ T cells, and PSMB9 is induced by interferon stimulation in SLE [41, 48]. All these core genes require additional studies to elucidate the complex interaction with clinical features.

**Table 1:** The previous studies about core genes in autoimmune disease

Gene	Tissue	Function	Author	DOI
GPB1	Blood	Promotes antimicrobial immunity and cell death. Key mediator of angiostatic effects of inflammation and is induced by interferon (IFN)-α and IFN-γ.	Liu, et al. [34]	10.1007/s10067-018-4138-7
CD36	Blood	Expresses on the cell surface of monocyte/macrophages and involved in the recognition and uptake of pro-atherogenic oxidized low-density lipoprotein (LDL).	Reiss, et al. [35]	10.3181/0806-BC-194
FCER1G	Spleen	Associated with multiple leukocyte receptor complexes and mediates signal transduction.	Sweet, et al. [36]	10.4049/jimmunol.1600861
CLEC7A	Blood	Involved in the clearance of apoptotic cells, uptake and presentation of cellular antigens and triggers different cytokines and chemokines.	Salazar-Aldrete, et al. [37]	10.1007/s10875-012-9821-x
ITGB2	Bone Marrow	Encodes integrin β2 protein (CD18). Plays important roles in leukocyte adhesion, immune and inflammatory reactions, immigration through endothelial and chemotaxis.	Zimmer, et al. [38]	10.1371/journal.pone.0013351
LILRB4	Blood	Associated with increased inflammatory cytokine levels in SLE and is expressed by many leukocytes.	Jensen, et al. [39]	10.1136/annrheumdis-2012-202024
HLA − DRA	Blood	SLE susceptibility genes and plays a central role in the immune system by presenting peptides derived from extracellular proteins.	Liu, et al. [40]	10.2174/1566524019666190424130809
PSMB9	Skin	Upregulates in the pathophysiology of cutaneous lesions of dermatomyositis and SLE.	Nakamura, et al. [41]	10.1111/bjd.14385
BTK	Blood	Plays an important role in both B cell and FcgammaR mediated myeloid cell activation. BTK inhibition may be a promising treatment approach for lupus nephritis.	Kong, et al. [42]	10.1007/s10067-017-3717-3
PYCARD	Blood	Forms inflammasome complexes mediate the inflammatory and apoptotic signaling pathways.	Shin, et al. [43]	10.1002/art.40672
CFP	Blood	The only positive regulator of the complement system. Recognized apoptotic and necrotic cells.	Cohen, et al. [44]	10.1002/path.2893
CFD	Blood	Encodes a protein functioned as an adipokine that involved in regulation of immune system and inflammatory responses.	Chougule, et al. [45]	10.1016/j.cyto.2018.08.002
MARCO	Blood	Binds to apoptotic cells and contribute to the clearance of apoptotic cells.	Chen, et al. [46]	10.1186/ar3230
CD3D	Blood	Single nucleotide polymorphism in the immune compartment and B cells, also involved in T cell signaling.	Lindén, et al. [47]	10.1186/s13293-017-0153-7
PSMB8	Blood	Involved in antigen-processing and presentation in naïve CD4 + T cells and hypomethylated in SLE.	Renauer, et al. [48]	10.1136/lupus-2015-000101

The current work is the first to use CIBERSORT to analyze immune cell infiltration of glomerular tissue in LN. All data were derived from GEO and were therefore reliable. The correlation results of CIBERSORT and GSEA to obtain core genes were validated in clinical data, leading to many new information for our future research. The analytical methods were scientific and novel. However, our study has some limitations. Only a few datasets of LN were available on the GEO database; therefore, the number of samples included in this study was relatively small. However, despite the small sample sizes, we still found some significant differences among groups. In addition, clinical tests need to be conducted to support our results.

## Conclusions

Our study provided a new insight into the immune filtration of LN. Five types of immune cells revealed important associations with LN, and monocytes showed the largest differences in the cellular composition of immune infiltration. Fifteen core genes that were related to clinical manifestation were analyzed. These genes may perform crucial functions, and further analysis of these genes in LN may identify targets for immunotherapy.

## Methods

### Microarray data processing

The data in our study came from a public domain. The normalized expression matrix and sample information were downloaded from the GEO database (www.ncbi.nlm.nih.gov/geo). We used “lupus nephritis” as a keyword for searching. The data selection criteria were as follows: (1) the study type was expression profiling by array; (2) the organisms must be *Homo sapiens*; (3) the samples of each dataset must include glomerular tissue. In accordance with the above criteria, the GSE32591 microarray dataset based on the Affymetrix Human GeneChip U133A (affy) platform was hit and adopted for CIBERSORT. The GSE113342 microarray dataset based on nCounter Nanostring Human Immunology v2 was used to demonstrate the association between selected genes and clinical feature later. Only 500 immune-related genes were detected in this dataset.

### Evaluation of immune cell infiltration

Gene expression datasets of GSE32591 were processed to remove the null values. The missing values were supplemented by KNN method in “impute” package [49], the format was prepared in accordance with the accepted format of CIBERSORT, and then data were uploaded to the CIBERSORT web portal (http://cibersort.stanford.edu/). We used the original CIBERSORT gene signature file LM22, which defines 22 immune cell subtypes, to analyze datasets from human glomerular tissues and normal tissues. CIBERSORT *p*-value < 0.05 was included.

### Differential analysis of immune cell infiltration types

To analyze the significant differential expression of different cell types of immune cells, we used the difference analysis between the disease group and the control group. Limma package and Bayesian method were used to construct a linear model [50]. *P*-value < 0.05 was the cut-off standard. To further understand the relationship between these different types of immune cell infiltration, Pearson correlation coefficient was used to find the correlation between these differentially expressed types of immune cells.

### GSEA preparation

GSEA is an analytical method for genome-wide expression profile microarray data. It can identify functional enrichment by comparing genes with predefined gene sets. A gene set is a group of genes that shares localization, pathways, functions, or other features. GSEA was conducted using clusterProfiler package (version 3.5) [51]. The fold change of gene expression between LN group and control group was calculated, and the gene list was generated according to the change of |log2FC|. Then, we utilized GSEA-based enriched Gene Ontology (GO) and Kyoto Encyclopedia of Genes and Genomes (KEGG) analyses.

#### GSEA-based enriched GO analysis

GO analysis includes three categories: molecular function, biological process, and cellular component. In the present study, we only selected biological process to perform GO analysis. GO analysis was performed through gseGO function in clusterProfiler package. The adjusted *p*-value < 0.05 was set as the cut-off criteria. The connections between the most significant GO terms and participating genes were visualized by GOenrich package with a network diagram.

#### GSEA-based KEGG pathway analysis

KEGG pathway enrichment analyses were also conducted by gseKEGG function in clusterProfiler package. The adjusted *p*-value < 0.05 was set as the cut-off criteria.

### Core gene list and correlation analysis

The core gene list obtained in the most significant GO term was analyzed by Spearman correlation with the differentially expressed immune cells from CIBERSORT results. Five groups of correlation analysis data were obtained. *P*-value < 0.05 was used as the cut-off standard, and genes with the top 10 highest absolute values of correlation coefficients were visualized in each group.

### Validation of core genes and association with clinical manifestations

In dataset GSE113342 with clinical information, patient part B was excluded because it was data after treatment, and only first renal biopsy data (patient part A), which had approximately 500 immune gene expression values that coincided with the genes obtained in the most significant GO term associated with immune response, were chosen for analysis. Gene intersection was calculated first, and the Spearman correlation analysis between these intersecting genes and clinical information, such as age, grade, and 12-month treatment response, was further applied.

## Supplementary information

**Additional file 1.** The details of GO analysis.

## Data Availability

The datasets in the current study come from CEO database: GSE32591 and GSE113342.

## Abbreviations

A2M: Alpha-2-Macroglobulin
BTK: Bruton Tyrosine Kinase
C1QA: Complement C1q A Chain
C1QB: Complement C1q B Chain
C3AR1: Complement C3a Receptor 1
C5AR1: Complement C5a Receptor 1
CCR1: C-C Motif Chemokine Receptor 1
CD36: CD36 Molecule
CD3D: CD3d Molecule
CFD: Complement Factor D
CFP: Complement Factor Properdin
CLEC7A: C-Type Lectin Domain Containing 7A
CLEC7A: C-Type Lectin Domain Containing 7A
CX3CR1: C-X3-C Motif Chemokine Receptor 1
FCER1G: Fc Fragment of IgE Receptor Ig
FCN1: Ficolin 1
GBP1: Glycoprotein Hormone Alpha 2
HCK: HCK Proto-Oncogene
HLA-DQB1: Major Histocompatibility Complex, Class II, DQ Beta 1
HLA-DRA: Major Histocompatibility Complex, Class II, DR Alpha
ISG15: ISG15 Ubiquitin Like Modifier
ITGB2: Integrin Subunit Beta 2
LILRB4: Leukocyte Immunoglobulin Like Receptor B4
LPXN: Leupaxin
LYN: LYN Proto-Oncogene
MARCO: Macrophage Receptor with Collagenous Structure
OAS1, OAS2, OAS3: 2′-5′-Oligoadenylate Synthetase 1, 2, 3
PLSCR1: Phospholipid Scramblase 1
PRKCH: Protein Kinase C Eta
PSMB8: Proteasome Subunit Beta 8
PSMB9: Proteasome Subunit Beta 9
PTPRC: Protein Tyrosine Phosphatase Receptor Type C
PYCARD: PYD And CARD Domain Containing
RSAD2: Radical S-Adenosyl Methionine Domain Containing 2
SYK: Spleen Associated Tyrosine Kinase
TLR2: Toll Like Receptor 2
